# The Structure Features and Improving Effects of Polysaccharide from *Astragalus membranaceus* on Antibiotic-Associated Diarrhea

**DOI:** 10.3390/antibiotics9010008

**Published:** 2019-12-23

**Authors:** Shanshan Li, Yuli Qi, Duoduo Ren, Di Qu, Yinshi Sun

**Affiliations:** Institute of Special Animal and Plant Sciences, Chinese Academy of Agricultural Sciences, Changchun 130112, China; lishanshan@caas.cn (S.L.); qiyuli521@163.com (Y.Q.); 15129289959@163.com (D.R.); qudi@caas.cn (D.Q.)

**Keywords:** *Astragalus membranaceus*, polysaccharide, antibiotic-associated diarrhea, gut microbiota, short-chain fatty acids

## Abstract

*Astragalus membranaceus* (*Astragalus*) is often used as a medical and food resource in China. The present study was designed to investigate the features and effects of polysaccharide from *Astragalus membranaceus* (WAP) on rats with antibiotic-associated diarrhea (AAD). WAP was mainly composed of glucose, galactose, arabinose and glacturonic acid, with glucan, arabinogalactan and RG-I regions, and it showed loosely irregular sheet conformation. WAP decreased the inflammatory cell infiltration of colon in AAD rats, increased propionate and butyrate production, improved metabolic levels, adjusted the diversity and composition of gut microbiota, increased the relative abundance of *Pseudomonas*, and decreased the relative abundance of *Allobaculum* and *Coprococcus*. In conclusion, WAP contained different types of polysaccharide regions and sheet three-dimensional conformation, while it ameliorated AAD by recovering the colon structure, adjusting the gut microbiota, and improving the SCFAs levels. The results can provide some data basis for natural products to alleviate the side effects related to antibiotics.

## 1. Introduction

Antibiotics are widely used in the world, saving countless lives and making great contributions to humanity. However, the side effects caused by nonstandard use of antibiotics have attracted more and more attention, especially diarrhea. Antibiotic-associated diarrhea (AAD) is a common phenomenon in antibiotic treatment [1], accompanied with changes of composition and diversity of gut microbiota, destruction of the gut structure, and dysbiosis of the gut environment, which might aggravate the ill process and is harmful for the recovery of patients.

Traditional Chinese medicine (TCM) has been widely used to treat many diseases as a supplemental or alternative medicine in Asian countries. *Astragalus membranaceus* (*Astragalus*) root, a plant commonly used in TCM, contains various bioactive compounds, such as flavones, saponins, and polysaccharides, which are always used as TCM to treat diarrhea. Scientific evidence shows that polysaccharides in *Astragalus* have multiple biological activities, including anti-cancer [2], immune-modulatory [3], anti-inflammatory [4], renal protective [5], antioxidant, antidiabetes, and cardioprotective activities [6,7]. It has been reported that polysaccharides from *Astragalus* (APS, MW, ~3.6 × 10^4^ Da) have α-(1→4)-D-glucan chain that contains one α-D-glucose at the C-6 position for every nine residues, which have shown anti-gastric cancer activity in rats [8]. Another report about the *Astragalus* polysaccharides APS-I and APS-II is composed of α-(1→3) glucose and 1→4, 1→6 glucose in the main chain, and with arabinose and xylose serving as the side chains; both APS-I and APS-II can inhibit tumor growth [9]. Other studies also reported that polysaccharides and oligosaccharides from TCM can alter the gut microbiota as well as maintain its homeostasis [10,11]. However, there are little reports on the effect of *Astragalus* polysaccharides in antibiotic-associated diarrhea.

In this study, we established an AAD rat model containing the composition and diversity of the gut microbiota, colon structure, short-chain fatty acid (SCFA) metabolites, and various metabolic processes. This model enables us to investigate the effects of *Astragalus* polysaccharides on AAD.

## 2. Materials and Methods

### 2.1. Materials

*Astragalus membranaceus* was collected from Fusong, Jilin, China and characterized by Professor Yinshi Sun. Lincomycin hydrochloride was purchased from CR Double-Crane Pharmaceuticals Co., Ltd. (Jinan, China). The TIANamp Stool DNA Kit (cat. No. DP328) was obtained from Tiangen Biotech Co., Ltd. (Beijing, China). Acetate, propionate, and butyrate were purchased from Sigma-Aldrich Co. (Darmstadt, Germany). All other chemicals and reagents were obtained from Sinopharm Group (Shanghai, China).

### 2.2. Extraction of Astragalus Polysaccharide

The dried roots of *Astragalus* (500 g) were suspended in 6 L of distilled water and heated at 100 °C for 3 h. The solution was cooled, filtrated, and the heating step was repeated. The solution was collected, concentrated to 1 L at 60 °C and centrifuged (4500 rpm, 10 min). Afterward, the solution was mixed with four volumes of anhydrous ethanol and centrifuged (4500 rpm, 10 min). The precipitates were collected and dissolved in 800 mL of distilled water, followed by an additional 3.2 L of anhydrous ethanol. The precipitates were dissolved in 800 mL distilled water, Sevag reagent (Chloroform: n-butyl alcohol = 4:1, v:v) was used three times to remove the protein layer, and then the precipitates were freeze-dried to yield Water-soluble *Astragalus* Polysaccharides (WAP).

### 2.3. Physiochemical Analysis of WAP

The concentrations of carbohydrate, uronic acid, and monosaccharide composition were determined as previously reported [12,13,14]. Protein was determined using a Dumas nitrogen analyzer (NDA 701) [15]. The molecular weight was estimated with high-performance gel permeation chromatography (HPGPC) on a TSK-gel G-3000PWXL column (7.8 mm × 300 mm, TOSOH, Tokyo, Japan) coupled with a Shimadzu HPLC system.

The FT-IR spectrum was recorded with a KBr pellet among wave lengths 500 and 4000 cm^−1^ on a NEXUS670 FT-IR spectrophotometer.

The ^13^C NMR spectrum was obtained on a Bruker AVIII spectrometer at 600 MHz. The sample (30 mg) was dissolved in D_2_O (1 mL, 99.8%), and the spectra were recorded at 25 °C. Acetone was used as an internal standard. 

WAP was added to a silicon plate with a thin-layer gold sputter-coated, and then synthesized hydro gel (CMH and NMH_3_) was freeze-dried and covered with gold before the analysis of scanning electron microscope (SEM). The three-dimensional structure of WAP was characterized on XL 30 ESEM (Philips).

### 2.4. Animals and Treatment

The present study was reviewed and approved by the Animal Care and Use Committee of the Institute of Special Animal and Plant Sciences, Chinese Academy of Agricultural Sciences (Ethical approval code: TCS2017021, January 2017). We used a total of 24 male Wistar rats (180 ± 20 g), which were purchased from Changsheng Laboratory Animal Technology Co., Ltd. (Beijing, China). Rats were maintained at a temperature of 22 ± 0.5 °C, a humidity of 50 ± 5%, and light: dark cycles of 12 h:12 h, and had free access to standard laboratory pellets and water. All animals were treated following the Guidelines for the Care and Use of Laboratory Animals recommended by the Institute of Special Animal and Plant Sciences, Chinese Academy of Agricultural Sciences and the Chinese Legislation on Laboratory Animals. The well-being of all animals was ensured throughout the study, and a minimal number of animals were used.

### 2.5. Experimental Design

The rats were acclimatized for 7 days and then randomly divided into four groups (*n* = 6/group): the control (C) group, antibiotic-associated diarrhea (DM) group, natural recovery (NR) group, and WAP treatment (WAP) group. To establish the ADD model, rats on the DM, NR, and WAP groups received lincomycin hydrochloride (10 mL/kg) twice a day for 4 days by gavage [16], whereas rats in the C group received an equivalent amount of physiological saline. The rats of the DM group were anesthetized with isoflurane using a small animal anesthesia machine. Blood samples were collected and centrifuged (1500 rpm, 10 min) to obtain the serum. Fecal contents (>0.5 g) were collected under sterile conditions and stored at −80 °C. Colon samples were fixed in 10% neutral formalin. Afterward, the rats were euthanized with CO_2_.

The rats of the WAP group continued to receive WAP (100 mg/kg) once a day for 7 days, whereas the rats of NR and C groups received an equivalent volume of physiological saline. After recovery, blood and colon samples, as well as fecal contents, were collected as described above.

### 2.6. Histological Analysis

Colon samples were fixed in 10% formalin, dehydrated in ethanol, embedded in paraffin, sectioned (4–5 μm), and stained with hematoxylin and eosin (HE). The sections were observed under an Olympus BH22 Microscope (Tokyo, Japan).

### 2.7. Microbiota Analysis

DNA extraction, PCR amplification of the V3–V4 regions of 16S rRNA genes, and sequence data analyses were performed as previously reported [17].

Operational taxonomic units (OTUs) were compared using the quantitative insights into microbial ecology (QIIME) platform, R software (ver. 3.2.0), and the Greengenes database [18]. Alpha diversity, including Chao 1 and Shannon indices, were calculated to compare the richness and diversity between groups [19,20]. Beta diversity was calculated using nonmetric multidimensional scaling (NMDS) to identify variations in the microbial communities [21]. Metastats Software was used to compare the abundances of taxa at phylum, class, order, family, and genus levels between samples or groups [22]. Phylogenetic investigation of communities by reconstruction of unobserved states (PICRUSt) analysis was used to identify the microbial communities based on high-quality sequences [23], in order to obtain the results of functional prediction of gut microbiota. All raw sequences were deposited into the NCBI Sequence Read Archives (SRP238192).

### 2.8. Measurement of SCFAs

The fecal contents of each rat were collected, and SCFAs were analyzed as previously reported [17]. The caecal content (100 mg) of each rat was placed into a centrifuge tube and then dissolved with 10 μL of 15% ortho-phosphoric acid, 100 μL of adipic acid (50 μg/mL, internal standard) solution and 400 μL of ether. The mixture was whirled for 1 minute and centrifuged (12,000 rpm/min, 10 min) at 4 °C, and then the supernatant was filtered through a 0.45 μm organic sample compatible membrane filter and used for the assay. Standard solutions of acetate, propionate and butyrate at different concentrations were prepared in ether. All assays were performed using the Agilent 6890N/5975BGC-MS System (Agilent, Santa Clara, CA, USA). The separation of each compound was achieved using an Agilent HP-INNOWAX capillary column (30 m × 0.25 mm, 0.25 μm). The initial oven temperature was 90 °C, which was maintained for 3 min and then increased to 120 °C by 10 °C/min, 150 °C by 5 °C/min, and finally 250 °C by 25 °C/min, then it was maintained for 2 min. The temperature of the ion source and injection port was set at 230 °C and 250 °C, respectively. One microliter solution was injected. The flow rate of helium was 1 mL/min with a 10:1 split ratio. Electron bombardment ionization (EI) source with a full scan and a sim scanning mode were adopted for mass spectroscopy, while the electron energy was 70 ev.

### 2.9. Statistical Analysis

All data were expressed as means ± standard deviation (S.D.). Statistical analyses were performed using Prism 5 Software. Comparisons between groups were performed using one-way analysis of variance (ANOVA) with Duncan’s range tests. Differences were considered significant when *p* < 0.05.

## 3. Results

### 3.1. Structure Analysis of WAP

#### 3.1.1. Monosaccharide Composition and Molecular Distribution

**Figure 1:**
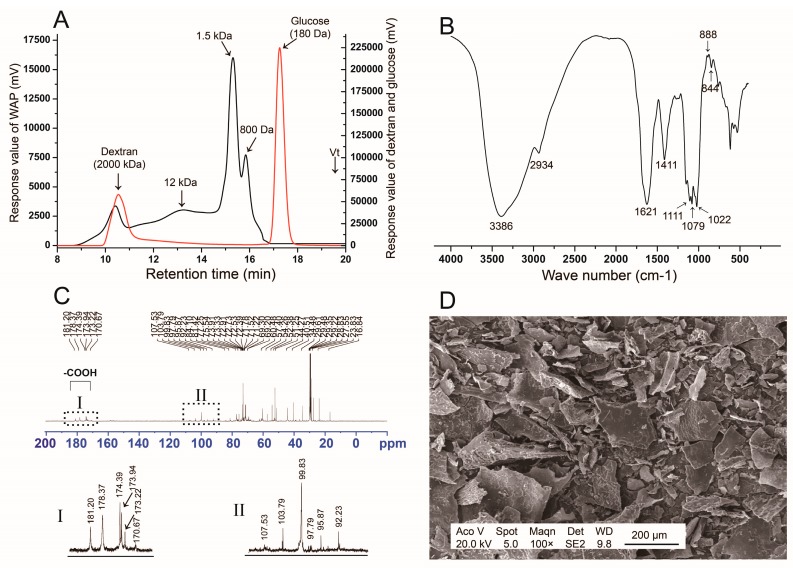
Structure analysis of WAP. (**A**) HPGPC profiles (Red line, dextran and glucose; Black line, WAP); (**B**) IR spectrum; (**C**) ^13^C NMR spectrum; (**D**) SEM analysis.

The yield of WAP was 4.5% (*w*/*w*). The total carbohydrate content was 96.0%, uronic acid content was 7.1%, and protein content was 0.85%. WAP was composed of glucose (80.9%), galactose (6.0%), arabinose (4.8%), galacturonic acid (6.7%), rhamnose (1.0%), and mannose (0.6%). The ratio of Rha/GalA was about 0.15, according to the concept of type I rhamonogalactoronan (RG-I), which suggested the existence of an RG-I and homogalacturonan (HG) region. WAP had a wide molecular distribution, which showed four main peaks of >2000 kDa, 12 kDa, 1.5 kDa, and 800 Da in HPGPC profiles (Figure 1A), respectively.

#### 3.1.2. FT-IR Spectrum

FT-IR spectrum of WAP was shown in Figure 1B. The -OH stretching vibration appeared at 3386 cm^−1^. The C-H stretching and deformation vibration were at 2934 cm^−1^ and 1411 cm^−1^, respectively. Peaks at 1111 cm^−1^, 1079 cm^−1^ and 1022 cm^−1^ represented the existence of pyran monosaccharide. The weak peaks near 888 cm^−1^ and 844 cm^−1^ indicated the existence of α-glucan or β-glucan residue. There were no peaks near 1260 cm^−1^ and 1730 cm^−1^, which indicated the absence of an acetyl group. 

#### 3.1.3. ^13^C NMR Analysis

^13^C NMR spectrum (Figure 1C) results showed that the anomeric carbon signals of 1,3,5-linked α-Araf, 1,3-linked α-Galp, and 1,4-linked α-Glcp were at 107.53, 103.79, and 99.83 ppm [15], respectively. The -CH_3_ of 1,2-linked α-Rhap was at 16.84 ppm. The -COOH of 1,4-linked GalA were at 170.67–181.2 ppm. These results suggested that WAP might be a mixture of glucan, arabinogalactan, and RG-I regions with the existence of 1,4-Glc, 1,3-Gal, 1,2-Rha, 1,4-GalA, and 1,3,5-Ara, which always appear in plant polysaccharides. The existance of 1,4-glucan was consistent with the polysaccharide fraction APS from astragalus reported previously [8].

#### 3.1.4. SEM Analysis

SEM analysis was carried out to identify the three-dimensional structure of WAP (Figure 1D). WAP showed a non-smooth surface and irregular sheet structure. Fragments of different sizes were loosely grouped together with flaky branches at the edges and on the thin sheet.

### 3.2. Normal Status of Rats

**Figure 2:**
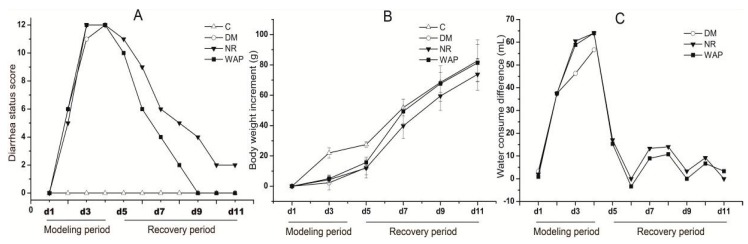
Effects of WAP on total diarrhea status scores (**A**), weight increment (**B**), the difference in water consumption (**C**). C, control group; DM, antibiotic-associated diarrhea group; NR, natural recovery group; and WAP, *Astragalus* polysaccharide group. Data are expressed as means ± S.D. (*n* = 6).

The rats with AAD showed lower food consumption, increased water intake, and higher defecation frequency, suggesting a successful establishment of the AAD rat model. A diarrhea status score was used to assess the severity of diarrhea according to the parameters in Table 1, and calculated by the sum of the six rats’ scores. Bodyweight increment was compared with the baseline on day 1, and the difference in the volume of water consumed was compared with the control group (Figure 2). Diarrhea status scores and the weight of rats gradually increased, and the difference in water consumption significantly increased during the establishment period of the model. Compared with physiological saline treatment, the diarrhea status scores and difference in water consumption were decreased; bodyweight increment was increased after treating with WAP. Therefore, WAP could better promote the normal status recovery of antibiotic-associated diarrhea in rats.

### 3.3. Effects of WAP on Colon Morphology

**Figure 3:**
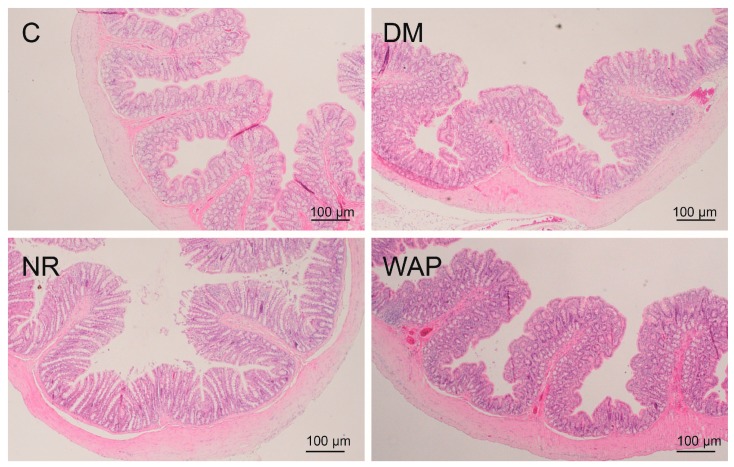
Colon structure (magnification, 40×) of the control group (C), antibiotic-associated diarrhea group (DM), natural recovery group (NR), and *Astragalus* polysaccharide group (WAP).

Hematoxylin and eosin staining were used to examine the morphology of the colon (Figure 3). The colons of rats in group C showed normal histological features, with smooth and uniform intestinal villi. No inflammatory infiltration was observed. However, the colons of rats in the DM group showed morphological damage, the intestinal villi were shorter, the mucosal surface was rough, and inflammatory infiltration with acidic granulocytes was observed. Compared to the NR group, the colons of rats in the WAP group showed marked morphological improvement; the intestinal villi were longer and denser, the mucosal surface was smoother, the intestinal epithelial cells were more compact, and the cell shedding and intestinal mucosal edema were reduced. The colon structure of the WAP group was closer to normal control than the NR group.

### 3.4. Effects of WAP on Gut Microbiota Dysbiosis

#### 3.4.1. α and β Diversity Analysis

**Figure 4:**
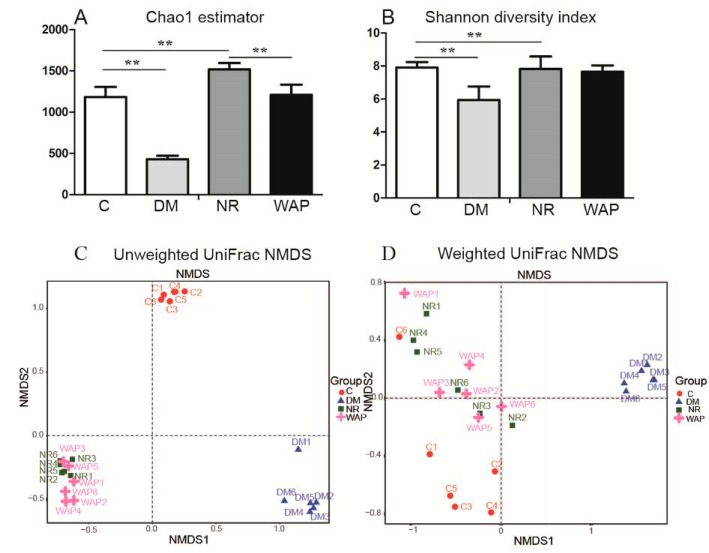
α and β diversity analysis of the gut microbiota. (**A**) Chao1 estimator; (**B**) Shannon diversity index; (**C**) unweighted UniFrac NMDS; (**D**) weighted UniFrac NMDS. C, control group; DM, antibiotic-associated diarrhea group; NR, natural recovery group; and WAP, *Astragalus* polysaccharide group. Data are expressed as means ± S.D. (*n* = 6). ** *p* < 0.01.

Chao 1 and Shannon diversity analyses were used to identifying differences in the richness and diversity of the gut microbiota. The results of Chao 1 analysis showed that the gut microbial richness was significantly decreased in rats of the DM group compared with those of group C. However, the gut microbial richness was restored in rats of groups NR and WAP (Figure 4A). There were significant differences between groups C and DM, groups C and NR, and groups NR and WAP. The results of Shannon analysis showed that the gut microbial diversity significantly decreased after antibiotic treatment compared with rats of group C, whereas no difference was observed between rats of groups NR and WAP (Figure 4B).

UniFrac NMDS was used for diversity analysis of OTU phylogenetic relationships to identify similarities in microbial communities between groups. Unweighted and weighted UniFrac NMDS was used to identify similarities in rare and dominant species, respectively, in rats of the four groups (Figure 4C,D). According to unweighted UniFrac NMDS, the microbial communities were distinct, with rats of the WAP and NR groups showing some similarities. According to the weighted UniFrac NMDS, the microbial communities were distinct in rats of the DM group; however, rats of C, NR, and WAP groups showed similarities. Compared with group C, there were differences in rare and dominant species in rats of the DM group, but similarities in richness and diversity in NR and WAP groups. These findings suggest that WAP restored the gut microbiota, especially the dominant species after antibiotic treatment.

#### 3.4.2. Composition Shifts of Key Microbial Phylotypes

**Figure 5:**
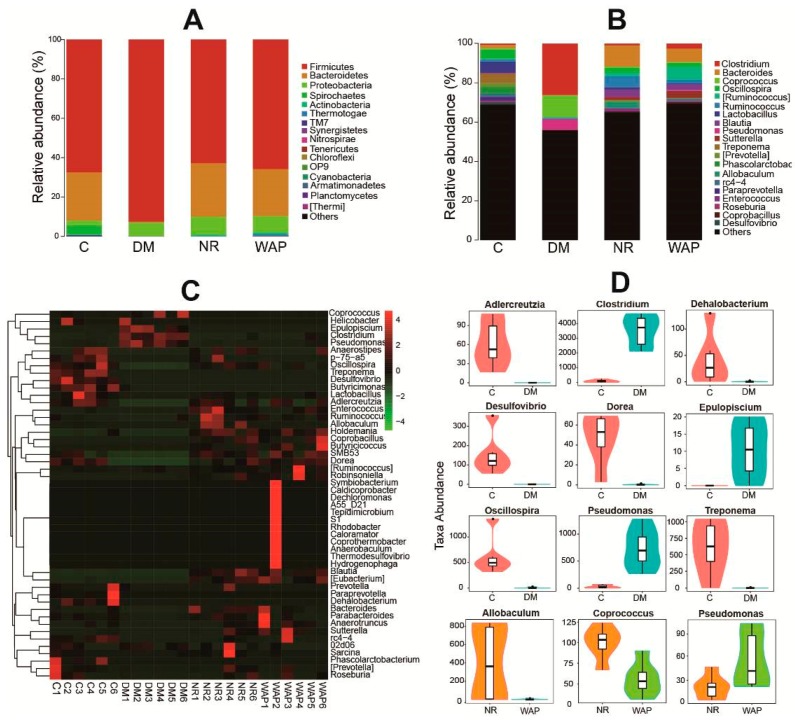
WAP altered the gut microbiota composition. (**A**) phyla level, (**B**) genus level, (**C**) heat map, (**D**) comparison between groups C and DM, and groups NR and WAP at the genus level. C, control group; DM, antibiotic-associated diarrhea group; NR, natural recovery group; and WAP, *Astragalus* polysaccharides group.

The histogram and heat map results showed significant shifts in the relative abundance of gut microbial species in groups C, DM, NR, and WAP (Figure 5A–C). At the phylum level, all the groups were mainly composed of Firmicutes, Bacteroidetes, and Proteobacteria (Figure 5A, Table 2). The relative abundance of Firmicutes was 78.7%, 92.4%, 62.8%, and 65.8% in groups C, DM, NR, and WAP, respectively. The relative abundance of Bacteroidetes was 24.5%, 0.5%, 27.2%, and 23.9% in groups C, DM, NR, and WAP, respectively. The relative abundance of Proteobacteria was 2.5%, 7.0%, 9.1%, and 8.4% in groups C, DM, NR, and WAP, respectively. Antibiotic treatment induced a significant decrease in the relative abundance of Bacteroidetes and a dramatic increase in Firmicutes and Proteobacteria. These changes in the gut microbial composition in the DM group compared to the C group suggested that a dysbiosis occurred. They also indicated a successful establishment of the diarrhea model. After treatment with physiological saline or WAP, the relative abundances of Firmicutes and Bacteroidetes returned to their respective basal levels, but Proteobacteria remained elevated. No significant difference in the main species at the phylum level was observed between WAP and NR groups.

Results of the heat map analysis showed differences in gut microbial richness at the genus level (Figure 5C). The gut microbiota of rats in group C was rich in *Oscillospira*, *Treponema*, *Desulfovibrio*, and *Butyricimonas*, whereas that of rats in group DM was rich in *Epulopiscium*, *Clostridium*, and *Pseudomonas*.

At the genus level, the composition of gut microbiota in groups DM, NR, and WAP showed a significant shift compared with group C (Figure 5B,D). The relative abundances of *Adlercreuzia*, *Dehalobacterium*, *Desulfovibrio*, *Dorea*, *Oscillospira*, and *Treponema* were decreased. However, the relative abundances of *Clostridium*, *Epulopiscium*, and *Pseudomonas* were increased in group DM compared to group C. After treating with physiological saline or WAP until the completion of the recovery period, the richness and composition recovered were comparable to group C, including the increase of *Adlercreuzia*, *Dorea*, and *Oscillospira,* and the decrease of *Epulopiscium* and *Pseudomonas*. 

Compared to group NR, the relative abundance of *Pseudomonas* was significantly increased, whereas those of *Allobaculum* and *Coprococcus* were decreased in the WAP group (Figure 5B,D). Although both physiological saline and WAP could decrease the abundance of *Pseudomonas* compared with AAD rats, WAP could not sufficiently reduce the abundance of *Pseudomonas*. However, WAP could effectively reduce the richness of *Allobaculum* and *Coprococcus* to improve the status of the gut environment compared with AAD rats. Therefore, WAP can regulate the composition and diversity of the gut microbiota to reduce diarrhea symptoms.

### 3.5. Impact of WAP on SCFA Production

**Figure 6:**
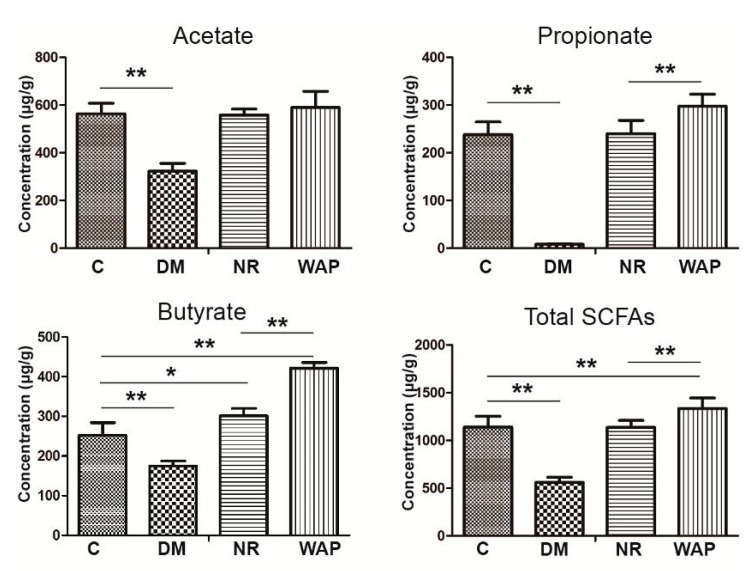
Effect of WAP on SFCA concentrations in fecal contents. C, control group; DM, antibiotic-associated diarrhea group; NR, natural recovery group; and WAP, *Astragalus* polysaccharide group. Data are expressed as means ± S.D. (*n* = 6). * *p* < 0.05; ** *p* < 0.01.

The effects of WAP on microbial metabolites were investigated by measuring the concentrations of acetate, propionate, butyrate, and total SCFAs (Figure 6). Rats of group DM showed significantly decreased concentrations of acetate, propionate, butyrate, and total SCFAs in the fecal samples compared with those of group C (*p* < 0.05). These states were recovered in rats belonging to the NR and WAP groups at different levels. Compared with group NR, the concentrations of propionate, butyrate, and total SCFAs were significantly increased in the WAP group (*p* < 0.05). However, there was no significant difference in acetate between the two groups.

### 3.6. Effect of WAP on the Functional Prediction of Gut Microbiota

**Figure 7:**
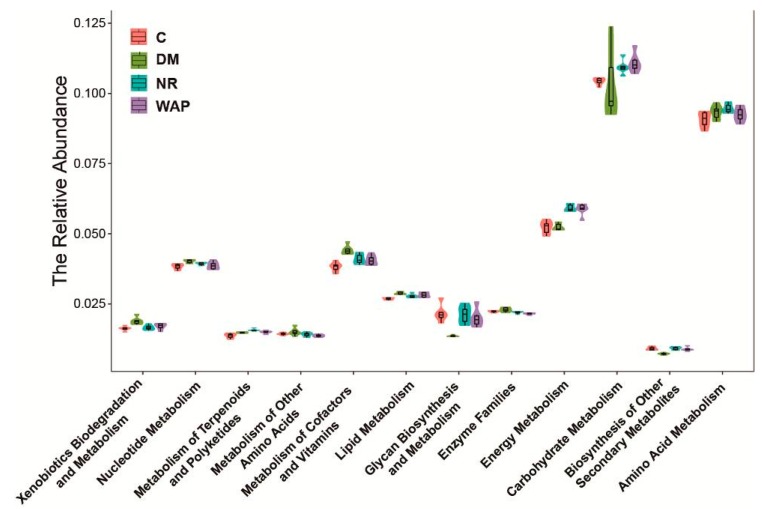
Effect of WAP on metabolic processes. C, control group; DM, antibiotic-associated diarrhea group; NR, natural recovery group; and WAP, *Astragalus* polysaccharides group.

As shown in Figure 7, AAD decreased the kinetics of carbohydrate and energy metabolic processes but increased the kinetics of amino acid metabolic processes. Energy metabolism was improved in rats belonging to the NR and WAP groups. Carbohydrate metabolic processes were enhanced, whereas amino acid metabolic processes were decreased in rats of the WAP group compared with those of the NR group.

## 4. Discussion

For the past 2000 years, plants in China have been used as herbal supplements and medicines to improve human health and treat various diseases [24]. The multiple uses, components, and targets of these plants have attracted the interest of investigators [25,26,27]. However, the active components of many plants remain unknown. The study of interactions between the active components of plants and gut microbiota has opened many new insights into the exploration and functions of natural resources. Several studies have focused on the effects of chemical components on the gut microbiota of the mammalian host in health and disease models [28,29,30]. Gut microbiota is important for host health since it affects several processes, including the host’s metabolism, shapes systemic immunity, maintains gastrointestinal tract homeostasis, and affects brain function and behavior. Gut microbiota can also metabolize various active components and produce metabolites with variable bioactivity or toxicity that can affect microbial communities in the gut. 

Polysaccharides are widely distributed in plants and have been reported to modulate the gut microbiota under various conditions [31]. *Polygonatum kingianum* polysaccharides can improve the gut microbial environment in diabetic rats [22]; *Ganoderma lucidum* polysaccharides can affect the gut microbial environment in mice with chronic pancreatitis [32]; *Panax ginseng* [33] polysaccharides can modulate the gut microbial environment in mice with antibiotic-associated diarrhea; and *Dictyophora indusiata* polysaccharides [34] can promote recovery from antibiotic-driven intestinal dysbiosis and improve gut epithelial barrier function in mice.

*Astragalus membranaceus* is used as a traditional medicinal plant in China. In this study, polysaccharides from *Astragalus membranaceus* were obtained, and one AAD rat model was established to investigate the effects of *Astragalus* polysaccharides on AAD. At the genus level, there was a significant decrease in the richness of *Adlercreuzia*, *Dehalobacterium*, *Desulfovibrio*, *Dorea*, *Oscillospira*, and *Treponema*, but an increase in the richness of *Clostridium*, *Epulopiscium*, and *Pseudomonas* in the DM group compared with the C group. WAP treatment could significantly improve the richness and diversity of the gut microbiota in AAD rats. Compared with the DM group, WAP increased the richness of Bacteroidetes and Proteobacteria but decreased the richness of Firmicutes, which suggested that WAP could restore microbial species at the phylum level. Compared to physiological saline treatment, the relative abundance of *Pseudomonas* was significantly increased, whereas that of *Allobaculum* and *Coprococcus* was decreased in the WAP group. Both physiological saline and WAP could reduce the abundance of *Pseudomonas* compared with AAD rats. However, WAP could reduce the richness of *Allobaculum* and *Coprococcus* to a level lower than in NR rats, and closer to that in C rats, which could maintain the healthy status of gut microbiota. It can be concluded that WAP can improve AAD by modulating the composition and diversity of the gut microbiota. Compared with the polysaccharides we have extracted from *Schisandra chinensis* [17], which could increase the relative abundance of *Blautia*, *Intestinibacter*, and *Lachnospiraceae*-*UCG*-*008*, but decrease the relative abundance of *Ruminococcus-1*, *Ruminococcaceae-UCG-014*, and *Erysipelatoclostridium* at the genus level, suggesting that different polysaccharides can alter the gut microbiota differently, which is possibly due to the variations of monosaccharide composition, fine structure, and space conformation. Although the mechanisms responsible for balancing the gut microbiota by WAP, WSP, or other polysaccharides from plant resources need more research, our results might offer some data and references on similar research.

The changes in the colon structure and SCFA concentrations were also associated with antibiotic treatment, and they reflected the status of the gut environment. In this study, rats of the DM group showed an abnormal colon structure and decreased concentrations of acetate, propionate, butyrate, and total SCFAs, suggesting that the AAD rat model was successfully established. Compared to the NR group, WAP could restore the colon structure and increase the concentrations of propionate, butyrate, and total SCFAs. These results indicated that WAP promoted a healthy gut structure and normal SCFA concentrations. Many studies have investigated the concentrations of SCFAs, which are derived from polysaccharides [31] and mainly composed of acetate, propionate, and butyrate. SCFAs have been reported to participate in the host metabolism as important signaling molecules [35]. They are also important to the gut microbial environment and closely involved in immune, anti-tumor, and anti-inflammatory activities [36,37]. Generally, SCFAs have beneficial effects on hosts. We found that WAP significantly improved the concentrations of propionate, butyrate, and total SCFAs in the caecum of treated rats compared to NR rats. These findings were different from those on *Schisandra chinensis* polysaccharides, which increased the concentrations of acetate, propionate, and total SCFAs. Therefore, variable polysaccharide structures can affect the fermentative activity of different bacteria in the intestinal tract to produce different SCFAs. Our results also indicate that WAP could improve the gut environment by producing SCFAs.

Gut dysbiosis is associated with many diseases and metabolic syndromes [38,39], such as diabetes, obesity, inflammatory bowel disease, fatty liver disease, and liver cirrhosis. Therefore, it is crucial to maintain a healthy gut microbial environment. Polysaccharides can alter the composition of the intestinal microbial community, and this phenomenon may offer new insights into the mechanisms involved and how natural active components can treat disease. Therefore, the purification of WAP was carried on in our research group in order to obtain a homogeneous polysaccharide fraction for further study of the underlying molecular mechanisms and structure-activity relationship.

## 5. Conclusions

In conclusion, WAP was mainly composed of glucose, galactose, arabinose, and glacturonic acid. There were glucan, arabinogalactan and RG-I regions in WAP, and it showed a loosely irregular sheet conformation. WAP exerts beneficial effects on rats with AAD by restoring the gut structure, improving the diversity, composition, and metabolic function of the gut microbiota, and increasing the production of SCFAs. Compared to rats of the NR group, WAP adjusted the relative abundance of *Pseudomonas*, *Allobaculum*, and *Coprococcus* at the genus level; significantly increased the concentrations of propionate, butyrate and total SCFAs; and recovered the metabolic processes of gut microbiota. These results indicate that WAP could serve as a potential natural product to ameliorated AAD, and the effects might be associated with WAP’s modulating effects on gut microbiota.

## Figures and Tables

**Table 1:** Diarrhea status assessment.

Scores	Diarrhea Status
0	Normal
1	Loose, light color, and nonstick perianal stool status; general mental state
2	Adhesion stool in the anus, mental depression, no appetite for food, weight loss

**Table 2:** The relative abundance of gut microbiota at the phylum level.

Relative Abundance (%)	Group			
	C	DM	NR	WAP
Firmicutes	78.7 ± 11.2 ^a^	92.4 ± 3.4 ^b^	62.8 ± 14.9 ^a^	65.8 ± 12.4 ^a^
Bacteroidetes	24.5 ± 14.4 ^a^	0.5 ± 0.7 ^b^	27.2 ± 13.7 ^a^	23.9 ± 13.6 ^a^
Proteobacteria	2.5 ± 1.0 ^b^	7.0 ± 3.5 ^a^	9.1 ± 3.1 ^a^	8.4 ± 4.1 ^a^

a,b Data within a column with different superscripts differed significantly (*p* < 0.05).
